# Balance control in aging: improvements in anticipatory postural adjustments and updating of internal models

**DOI:** 10.1186/s12877-015-0161-6

**Published:** 2015-12-07

**Authors:** Alexandre Kubicki, France Mourey, François Bonnetblanc

**Affiliations:** Institut National de la Santé et de la Recherche Médicale (INSERM), Unité 1093, Cognition, Action et Plasticité Sensorimotrice, Campus Universitaire, Université de Bourgogne, BP 27877, F-21078 Dijon, France; UFR STAPS, Université de Bourgogne, BP 27877, F-21078 Dijon, France; UFR Santé, Université de Bourgogne, 7 Boulevard Jeanne d’Arc, 21000 Dijon, France; INRIA, LIRMM, équipe DEMAR, Université de Montpellier 2, 34095 Montpellier, France; Institut Universitaire de France, Paris, France

**Keywords:** Geriatric rehabilitation, Balance function, Anticipatory postural adjustments

## Abstract

Postural stability of older subjects can be estimated during orthostatic equilibrium. However, dynamic equilibrium is also important to investigate risks of fall. It implies different interpretations of measures given by force plates. Same dependant variables (e.g. center of pressure displacement) cannot be interpreted the same ways depending of the type of equilibrium that is investigated. In particular, sways increases during dynamic equilibrium and before movement execution may reflect an improvement of feedforward control.

## Correspondence

In a recent article published in BMC Geriatrics, Schoene et al. [1] conducted a literature review about the effects of cognitive-motor training in reducing falls for aged individuals. Their study gives a global picture of rehabilitation in the field of Geriatrics with an emphasis on balance control. It is urgent to promote such studies given the current demographic context. Indeed, as highlighted in a recent work [2], the population aging is an international phenomenon that will continue during several decades. To illustrate this process, S. Harper reported in this paper that “by 2050, there will be the same number of old as young in the world, with 2 billion people aged 60 or over and another 2 billion under age 15”.

On the basis of a large literature review, Schoene and colleagues reported that sway increases can be considered as a non-optimal balance control. This viewpoint, shared by several researchers [3–6], may however be discussed and modulated depending on the context of balance assessments.

In physics, a system can be at stable or unstable equilibrium. At stable equilibrium the displacements of the centre of gravity can be very large without falling under the influence of another attractor (e.g. a ball in a concave recipient). Considering that the biomechanics of orthostatic posture can be conceptualized as an inverse pendulum, it is generally advocated that larger displacements of the center of pressure put the system at higher risk of falling, as acknowledged in the literature [3–6]. This latter assumption may however not apply directly to dynamic equilibrium, which is mainly involved in falls during aging [7] and corresponds to self-generated perturbations of balance.

During dynamic equilibrium (and not in orthostatic stance), sways increase can occur ahead of any movement. These anticipatory postural adjustments (APA) are highly important because they reveal the capacity of the central nervous system to anticipate the perturbation associated with the upcoming movement and to compensate for it [8]. In other words, these are illustrations of estimates and predictions of physical and sensory consequences of the movement: i.e. the so-called internal feedforward (or predictive) models [9]. These models are critical in motor control since they allow compensating for transduction, transmission and processing biophysical delays [10, 11]. As a consequence, the decrease with aging of these prior displacements of the center of pressure (with respect to the onset of the focal movements), suggests that such individuals are not able to anticipate for the perturbations generated by the displacement of the limb and to counter for its destabilizing effects. Instead, they react afterwards: they behave more in a reactive rather than in a predictive manner [12]. This decrease of the anticipatory activity can be recovered following training, suggesting that an update of internal predictive models is possible in the case of normal aging [13] and for frail elderly individuals [14]. In this approach, it is believed that a vicious circle is involved with aging: a decrease in the overall motor activity could yield a default in the update of internal models, that is constantly required to perform fast and accurate movements [12–14].

A key issue concerns the duration of the effects obtained with neurorehabilitation exercises specifically targeting the update of feedforward models. This latter is possible through the repetition of movements in what is called sensorimotor adaptation. In this process the motor command is modified quite rapidly from one trial to another. However, adaptation generally induces short-term improvements that may not last very long. In addition, targeting this adaptation during neurorehabilitation could be limited and may not be true motor learning (i.e. learning a new motor sequence rather than adapting an existing one to a new context) [15]. For frail individuals, the decrease of spontaneous motor activity may be a critical problem for sensorimotor adaptation, the update of internal models and true motor learning (of a new motor sequence). Interestingly, however, in frail individuals we observed some improvements of APA that persisted between separated sessions of practice for several weeks [14].

To conclude, a sways increase during dynamic equilibrium and before movement execution may reflect an improvement of APA, suggesting an update of internal models. These effects can be observed during several weeks of a specific training.
